# Supplementary material for: The Systems Biology Research Tool: evolvable open-source software
Source: BMC Syst Biol. 2008 Jun 29;2:55. doi: 10.1186/1752-0509-2-55 (PMC2446383; doi:10.1186/1752-0509-2-55)
Supplement: Additional file 1 — SBRT Archive. An archive of the current version of the Systems Biology Research Tool. [file 1752-0509-2-55-S1.zip › sbrt-1.4.0/doc/developers_guide/api/sbrt/kernel/fba/comp/class-use/FbaOptComputer.html]

Uses of Class sbrt.kernel.fba.comp.FbaOptComputer


|  |  |  |
| --- | --- | --- |
| **Overview** \| **Package** \| **Class** \| **Use** \| **Tree** \| **Deprecated** \| **Index** \| **Help** | |  |
| PREV   NEXT | **FRAMES**    **NO FRAMES**     **All Classes** |  |


---


## **Uses of Class sbrt.kernel.fba.comp.FbaOptComputer**

| Packages that use FbaOptComputer | |
| --- | --- |
| **sbrt.kernel.fba.comp** | Provides classes and interfaces for performing Flux Balance Analysis computations. |
| **sbrt.kernel.fba.proc** | Provides classes and interfaces for executing Flux Balance Analysis processes. |
| **sbrt.shell.mng.fba** | Provides classes and interfaces for creating Flux Balance Analysis process managers. |

Uses of FbaOptComputer in sbrt.kernel.fba.comp

Constructors in sbrt.kernel.fba.comp with parameters of type FbaOptComputer

`AbstractFluxIntervalComputer(FbaOptComputer computer)`
  
          Constructs a new flux interval computer with the default zero cutoff.

`AbstractFluxIntervalComputer(FbaOptComputer computer,
double zeroCutoff)`
  
          Constructs a new flux interval computer with the provided zero cutoff.

`FastMsFluxRangeComputer(FbaOptComputer computer,
java.util.Map<java.lang.String,java.util.Set<LinearComb<java.lang.String>>> fluxCaps,
LinearSystemSolution<java.lang.String> systemSolution,
double zeroCutoff)`
  
          Constructs a new flux interval computer.

`InitialPointComputer(FbaOptComputer computer)`
  
          Constructs a new initial point, or flux vector, computer.

`MsFluxIntervalComputer(FbaOptComputer computer,
java.util.Map<java.lang.String,java.util.Set<LinearComb<java.lang.String>>> fluxCaps)`
  
          Constructs a new Mahadevan-Schilling flux interval computer.

`MsFluxIntervalComputer(FbaOptComputer computer,
java.util.Map<java.lang.String,java.util.Set<LinearComb<java.lang.String>>> fluxCaps,
double zeroCutoff)`
  
          Constructs a new Mahadevan-Schilling flux interval computer.

`ReverseRxnFluxIntervalComputer(FbaOptComputer computer,
double zeroCutoff)`
  
          Constructs a new flux interval computer.

`RxnDeletionComputer(FbaOptComputer computer)`
  
          Constructs a new reaction deletion computer.

`SimpleFluxIntervalComputer(FbaOptComputer computer,
double zeroCutoff)`
  
          Constructs a new flux interval computer.

Uses of FbaOptComputer in sbrt.kernel.fba.proc

Constructors in sbrt.kernel.fba.proc with parameters of type FbaOptComputer

`CvFbaOptimizationExperiment(FbaOptComputer computer)`
  
          Constructs a new constraint-variation FBA optimization experiment.

`CvObjectiveFunctionProcess(FbaOptComputer computer)`
  
          Constructs a new constraint-variation objective function experiment.

`FbaOptimizationProcess(FbaOptComputer computer)`
  
          Constructs a new FBA optimization experiment.

`ObjectiveFunctionAnalysis(FbaOptComputer computer)`
  
          Constructs a new objective function experiment.

`StoichVariationProcess(FbaOptComputer computer)`
  
          Constructs a new constraint-variation FBA optimization process.

Uses of FbaOptComputer in sbrt.shell.mng.fba

| Methods in sbrt.shell.mng.fba that return FbaOptComputer | |
| --- | --- |
| `static FbaOptComputer` | `FbaOptManager.getComputer(ManagerInput<java.lang.String,java.lang.String> input, java.io.PrintWriter out)`             Creates and returns an FBA optimization computer based on the provided input. |
| `static FbaOptComputer` | `InitialPointManager.getComputer(ManagerInput<java.lang.String,java.lang.String> input, java.io.PrintWriter out)`             Creates and returns an FBA optimization computer based on the provided input. |

---


|  |  |  |
| --- | --- | --- |
| **Overview** \| **Package** \| **Class** \| **Use** \| **Tree** \| **Deprecated** \| **Index** \| **Help** | |  |
| PREV   NEXT | **FRAMES**    **NO FRAMES**     **All Classes** |  |


---
